# Is There an Association between Herpetic Infections and Giant Cell Arteritis? A Population-Based Study

**DOI:** 10.3390/jcm10010063

**Published:** 2020-12-27

**Authors:** Dong-ho Lee, Alfonso Iovieno, Claire A. Sheldon

**Affiliations:** Department of Ophthalmology and Visual Sciences, University of British Columbia, Vancouver, BC V5Z 3N9, Canada; alfonsoiovieno@hotmail.com (A.I.); claire.a.sheldon@gmail.com (C.A.S.)

**Keywords:** giant cell arteritis, temporal arteritis, herpes zoster, herpes simplex

## Abstract

Recent data suggests that herpes zoster (HZ) and herpes simplex virus (HSV) may be one of the underlying immunological triggers for giant cell arteritis (GCA). However, there is limited population-based data to support this. Our goal was to determine if herpetic infections increase the likelihood of GCA in the British Columbia (BC) population. The background prevalence of GCA was compared to the prevalence of GCA in subjects with HZ and HSV using diagnostic billing code data from an online BC database (BC Data Scout^TM^). BC residents ≥30 years old at the time of diagnosis from January 2000 to January 2019 were included. The relevant International Classification of Disease codes was used to identify patients with GCA, HZ, and HSV. Comparisons were made using two-sample Z tests. There were 4315 GCA diagnoses, from a total population of 3,026,005 subjects. The prevalence of GCA was 143 per 100,000 people. In terms of herpetic infections, 850 GCA cases were identified in 249,900 subjects with HZ versus 310 diagnoses of GCA in 163,170 subjects with HSV. The prevalence of GCA in subjects with HZ (0.340%) was significantly higher than the prevalence of GCA (0.143%) in the general population (*p* < 0.00001). The prevalence of GCA in HSV subjects (0.190%) was also significantly higher (*p* < 0.00001) than the population prevalence but lower than (*p* < 0.00001) the GCA with HZ prevalence. The likelihood of GCA appears to increase with herpetic infections, more significantly with HZ.

## 1. Introduction

Giant cell arteritis (GCA), also known as temporal arteritis, is a chronic granulomatous medium and large vessel vasculitis which can have devastating vision and life threatening consequences [1]. The exact pathophysiology of GCA remains uncertain, hindering the development of therapeutic options [1,2]. There is thought to be contributions from both genetic predispositions and environmental triggers which lead to a T-cell and macrophage mediated immune response to antigens within the pathogenic arteries [1]. Hypothesized causal agents have ranged from aging-related epigenetic factors to infectious sources such as *Chlamydia pneumoniae*, cytomegalovirus, or parvovirus [1,2,3]. Relatively recently, histopathological data has emerged which has suggested herpetic infections may be potential triggers, specifically varicella zoster virus (VZV) [4,5,6], the causative agent of chicken pox and herpes zoster (HZ), and herpes simplex virus (HSV) [7]. Antigens and DNA for VZV and HSV were found in GCA positive temporal artery biopsies in multiple studies [5,6,7,8], although more convincingly in the case of VZV than HSV. However, these studies remain inconclusive as there are similar studies which show little to no relationship to the same agents [8,9,10,11,12]. Furthermore, one study hypothesized that the previous positive VZV studies that were identified by the same research group may be due to non-specific background immunostaining rather than a true positive finding [13].

Outside of these histopathological analyses, there is limited population-based studies evaluating this hypothesis. One recent study from the UK highlighted a possible relationship with multiple infections and to a lesser degree, HZ, and GCA [14]. Another study from the United States reported an increased risk of GCA with recent HZ diagnosis [15]. Due to the large geographical variability in GCA prevalence [1,16], further research is necessary. Our goal was to use population-level data from a medical billing code database from British Columbia (BC), Canada, to determine if HZ or HSV infection correlates with GCA.

## 2. Experimental Section

We conducted a comparative descriptive study using a population database analysis. Research protocols were reviewed and approved by the Research Ethics Board of the University of British Columbia. Data collection and analysis were in adherence with the guidelines of the Declaration of Helsinki. An online British Columbia (BC) database, BC Data Scout^TM^ (Population Data BC, https://www.popdata.bc.ca/, report generated 11 September 2019, report ID 474094) [17], was used to extract diagnostic billing code data submitted to the BC government from physicians. As BC is a public single-payer healthcare system, these billing codes should encompass nearly all physician–patient interactions within BC. To identify cases of GCA, the relevant International Classification of Disease (ICD) was used (ICD-9: 4465). To account for cases of GCA which were not specifically billed as a GCA diagnosis, we combined codes for ischemic optic neuropathy (ICD-9: 3771, 3772, 3773, and 3774, which are codes for optic atrophy, other disorders of optic disc, optic neuritis, and other disorders of optic nerve, respectively) or retinal arteriolar occlusion (ICD-9: 3623, 3628, and 3629, for retinal vascular occlusion, other retinal disorders, and unspecified retinal disorders, respectively) with a concurrent procedure code for temporal artery biopsy (07025). To identify cases of herpes zoster (HZ) or herpes simplex virus (HSV), the relevant ICD codes were used (ICD-9: 053 and 054, respectively).

Data extraction included all BC residents ≥30 years old by 1 January 2000. This range was selected to include patients diagnosed with HSV, HZ, or GCA. Diagnoses made from 1 January 2000 to 31 January 2019 were included. The background prevalence of GCA in this population was compared to the prevalence of GCA in subjects with HZ and HSV. All comparisons were made using two-sample Z tests using Microsoft Excel 2013 and the significance level was set via the simple Bonferroni correction for multiple comparisons to α = 0.025. The effect of gender on the diagnosis of GCA, HSV and HZ was also investigated using a separate 2-by-2 Chi-Square test for each diagnosis compared to the background population. The significance level was set as α = 0.0125 via the simple Bonferroni correction.

## 3. Results

The population-level data extracted from BC Data Scout^TM^ showed a total population of 3,026,005 British Columbia (BC) residents who were ≥30 years old by January 1, 2000. There were 4315 probable giant cell arteritis (GCA) diagnoses within this group. The prevalence of GCA was 143 per 100,000 population ≥30 years of age. Using only the diagnostic code for GCA (excluding the temporal artery biopsy related codes discussed above), the prevalence was very similar, at 136 per 100,000. To determine if there were any GCA cases before the age of 50, which would be uncommon [1], we extracted billing data for patients older than 50 at the time of GCA diagnosis for comparison. As expected, the number of GCA diagnoses were the same, illustrating that all patients were diagnosed with GCA after 50 years of age. The prevalence of HZ was 82 per 1000 people and for HSV was 53 per 1000 people. Of the HZ diagnoses, 44.7% of them occurred between the ages 30 and 50, while for HSV, it was 68.4%. The demographic information available from the data extraction report is summarized in Table 1. There was a small yet significant (*p* < 0.00001) predominance of females in each of the study groups when compared separately to the background gender distribution.

**Figure 1:**
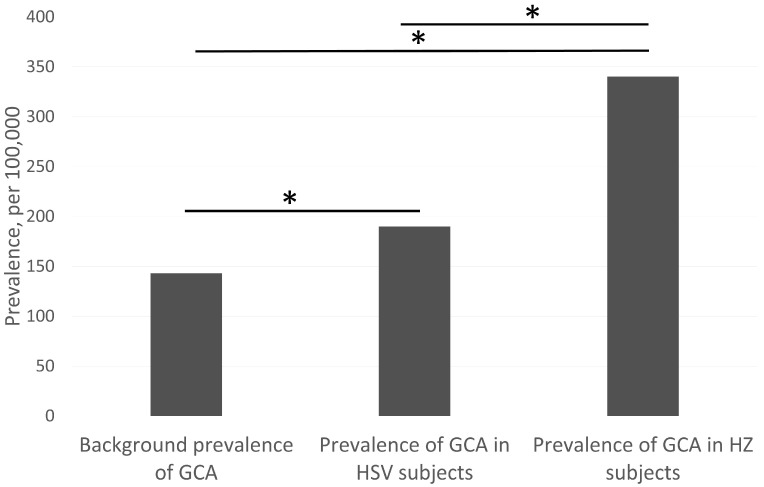
Comparison of the prevalence of giant cell arteritis (GCA) in British Columbia (BC) of the general population, patients with previous herpes simplex virus (HSV) and previous herpes zoster (HZ). Includes BC residents ≥30 years of age at the time of diagnosis. * represent significant differences at *p* < 0.00001.

In terms of subjects with herpetic infections and GCA, 850 GCA cases were found among 249,900 subjects with previous herpes zoster (HZ) as compared with 310 diagnoses of GCA in 163,170 subjects with previous herpes simplex virus (HSV). The prevalence of GCA in subjects with previous HZ (340 per 100,000 population) was significantly higher than the prevalence of GCA in the general population (*p* < 0.00001; Figure 1). The prevalence of GCA in HSV subjects (190 per 100,000 population) was also significantly higher (*p* < 0.00001) than the population prevalence of GCA, but lower than (*p* < 0.00001) the GCA with HZ prevalence (Figure 1).

## 4. Discussions

A growing number of studies suggest giant cell arteritis (GCA) is triggered by an infectious source [2,3]. The evidence ranges from the seasonal variability of the disorder, the preceding clinical symptoms of fever and malaise, the involvement of dendritic cells, T cells and interferon gamma, as well as the histopathological correlation with infectious agents [4,11,16,18]. Relatively recent studies have suggested herpetic infections may be one of the main contributory sources [4,5,6,7]. In our study, the prevalence of GCA was significantly higher in herpes zoster (HZ) patients than herpes simplex virus (HSV) patients and higher in both HZ and HSV patients than the background population (Figure 1). Our findings further support the hypothesis that herpetic infections may increase the likelihood of having GCA. Indeed, the histopathological evidence for a relationship between herpetic infections and GCA is stronger in HZ [4,5,6] than HSV [7]. This is in keeping with our finding that although HSV appears to increase the likelihood of GCA, HZ increases the likelihood even further. Although the exact mechanism of the link between herpetic infections and GCA need to be further elucidated if it exists, the suspected hypothesis is through a molecular mimicry mechanism, which induces an autoimmune T-cell reaction [19]. However, in recent small studies, there was no difference or possibly a negative correlation between the number of VZV targeted T-cells in GCA subjects as compared with controls [20,21]. This suggests other immunological factors may be at play instead.

Overall, we identified a GCA prevalence of 143 per 100,000 people ≥50 years of age. Although this is higher than previous studies which generally ranged from 1 to 30 cases per 100,000 people [1], it is less than the highest reported rate of 278 cases per 100,000 people [16]. A previous study in Saskatoon, Canada reported a lower prevalence of 9.4 per 100,000 [22], although this study was completed in a region with a relatively high proportion of aboriginal people, wherein GCA is thought to be less common [22]. The variability in prevalence likely represents the diversity in the genetic and environmental conditions of each study as most high prevalent areas appear to be in northern latitude countries or areas with strong Scandinavian lineage [1,16]. Our study similarly takes place in a relatively northern latitude region and Scandinavian heritage may contribute to our results as approximately 7.5% of the BC population ethnically originates from that region [23]. This study also supports the female predominance of GCA. With respect to GCA, we found that approximately 2.25 women were diagnosed with GCA for every man, similar to previous studies in populations of Northern European descent where it was approximately 2.5 to 1 [1,24]. This predominance appears to be less identifiable in Middle Eastern and Mediterranean countries [1]. The explanation for this sex difference is unclear but it may point towards the current pathophysiologic hypothesis which involves an immunological trigger and immune system overactivation [1,2,3,4]. Interestingly, a large scale autopsy study suggested prevalence estimates may be underestimating a true number closer to 1 in 100 people [25]. This variability and discrepancy also highlight the difficulty in studying GCA due to its large clinical variability and diagnostic challenges [1,18,26].

Our study also found the prevalence for HZ and HSV, 82 per 1000 people and 53 per 1000 people, respectively, with a female predominance of approximately 1:2 in HSV and 2:3 in HZ. Previous studies have reported varying numbers for the prevalence of HZ from 80 per 1000 people to 238 per 1000 [27,28,29,30]. As HZ is known to be more common with increasing age, some of this variability is due to the different age groups studied in different reports [27,28,29]. Our prevalence number may be on the lower end of previously reported numbers because we included people older than 30 years, which is younger than the age where most other studies identify HZ patients. Furthermore, the prevalence varies for different races [28,29]. Previous studies found a similar female predominance to our study for HZ [27,29,30], and this appeared to vary with age [27]. For HSV, previous reported prevalence values vary greatly depending on whether they assess seroprevalence versus clinical HSV and their geographic location. For seroprevalence of HSV antibodies, rates range from 9% to 80% of the population for HSV-1 with a female predominance [31,32,33,34,35,36]. For clinical HSV, there are limited results available. One study found 26 per 1000 people reported genital herpes [32] but this ignores the large number of HSV infections resulting in oral herpes. As our investigation used billing code diagnostic data from direct healthcare interactions, it is expected that our prevalence results are closer to that of rates described for all clinically significant HSV infections. Given that HSV infections do not always necessitate clinic visits, the true prevalence of symptomatic HSV is likely to be higher in our population than we report. This suggests that our data refers to clinically significant HSV, requiring physician assessment. Thus, perhaps clinically severe HSV infections may be the key correlate with GCA.

After considering the relationships and prevalence data, it cannot be ignored that many histopathological studies did not find HZ or HSV correlations in their studies [8,9,10,11,12]. Although some of these findings may be due to the small sample sizes or inadequate sensitivity in the laboratory tests, the more likely explanation is that there are numerous immunological triggers to GCA. One could argue that a large population study such as ours would have higher sensitivity for a relationship between herpetic infections and GCA than smaller histopathological studies, and thus more definitive, but there are previous large population studies which did not find a strong relationship with HZ and GCA [14,15]. This may be because these studies were based in the United Kingdom and United States, which would have different geographic and ethnic variables than our current study. Therefore, perhaps herpetic infections are only a major contributing factor for GCA in specific populations. Of note, the England et al. study found 5942 GCA cases in 16,686,345 subjects [15], which was a significantly different prevalence rate of GCA to our findings given the number of GCA cases was similar to our study but we had one fifth the number of total subjects. This further highlights the geographic inconsistency of this disorder. The variability of GCA is supported by the fact that some histopathological studies have shown a rather convincing proportion of HZ positivity in their GCA subjects [4,5,6], whereas others have shown a convincing absence of HZ [9,11]. However, another explanation for the discrepancy in histopathological studies is the variability in the protocols amongst these studies. The larger the number of sections stained per biopsy, the more often HZ was identified [4], which suggested it may be due to the skip-lesion nature of the disease process which produces the negative studies. In addition, a recent study has suggested that the previous studies that found a strong correlation with VZV in GCA, which were completed by one research group, may be from false positive background immunostaining [13].

There are relevant weaknesses in our study. First, there is lack of detail about our patient cohorts and temporality. As compared with other epidemiological and population-based studies, the available data we were able to extract from our database was limited. This made it impossible to complete age-matched or cohort-matched analyses used in other studies [14], and limited our analysis to simply comparing the prevalence. This introduces the possibility of other explanatory variables which may be increasing the prevalence of both GCA and herpetic infections which we could not foresee or control, such as ethnicity.

Furthermore, it was not possible to extract data on subjects diagnosed with GCA only after having had HZ or HSV as BC Data Scout did not release this type of temporal information. We suggest that, given the relative ages of common diagnosis, there is a presumption that HZ and HSV was diagnosed prior to GCA. From the available dataset, we were able to confirm at least 44.7% of the HZ and 68.4% of the HSV diagnoses occurred prior to the GCA diagnosis. We recognize weaknesses to our assumptions. First, if HZ and HSV indeed occurred prior to the diagnosis of GCA, this could be many years earlier to the GCA occurrence, especially given the high seroprevalence of both in elderly populations [27,36]. This would be less suggestive of the previously proposed molecular mimicry mechanism [19] as that should result in GCA relatively soon after a re-activation or infection with the virus, which is when the pathologic T cells would be activated. Second, if the diagnostic codes for HZ and HSV were generated at an older age, it is possible that the diagnosis occurred at the time of, or following, the diagnosis of GCA. Indeed, herpetic infections may occur during the immunosuppressive treatment of GCA. In this case, these patients may have had a preclinical, latent, or mild infection by the virus beforehand. For example, a mild infection only treated via over-the-counter medications or home remedies would not result in a billing code submission by a healthcare provider. The majority of HZ and HSV clinical presentations in an older age group would be a secondary infection or reactivation of the virus. Therefore, even if a portion of the diagnostic codes for HZ and HSV were from older individuals, with or without immunosuppression, it remains probable that the patient had a previous VZV or HSV infection prior to the onset of GCA. Even with the diagnosis of GCA prior to HZ or HSV, the two may still be linked. However, we would like to emphasize the lack of temporality in our data makes it difficult to identify causality between GCA and herpetic infections. Lastly, subjects with ischemic optic neuropathy or retinal artery occlusions with a temporal artery biopsy do not necessarily have GCA. However, this was only a small proportion of the overall number of GCA subjects in our study (340 subjects out of 4315).

Taking these weaknesses into account, our study has several strengths and findings that are valuable for moving our current knowledge forward. Our study is the first and largest epidemiological study, to date, to identify a link between HSV and GCA, whereas previous studies have focused only on VZV [14,15]. Furthermore, we have completed the first epidemiological examination of the link between GCA and herpetic infections in Canada, which is an important addition to the field due to the geographical variability of GCA [1,16]. Lastly, we have identified a statistically strong correlation in the main finding of our study, suggestive of a true relationship between GCA and herpetic infections.

Overall, our study suggests the likelihood of developing GCA seems to increase with herpetic infections, especially HZ. Further study is required to identify an exact causative relationship between the two. If a link is identified, the therapeutic implications of using antivirals for treatment and prevention of GCA would be an exciting avenue for future clinical research.

## Figures and Tables

**Table 1:** Demographic summary of study population as available from BC Data Scout^TM^.

		Age Ranges		Gender		
Cohort Group	Total Number	30–64	65+	Male	Female	Unknown
**All patients**	3,026,005	1,410,370	1,615,635	1,484,700	1,539,970	1335
**Patients with GCA**	4115	0	4115	1265	2850 *	0
**Patients with ION/RAO and TABx**	340	0	340	110	225 *	5
**Patients with HSV**	163,170	94,220	68,950	56,450	106,715 *	5
**Patients with HZ**	249,900	165,010	84,865	101,000	148,870 *	30

GCA, giant cell arteritis; ION, ischemic optic neuropathy; RAO, retinal artery occlusion; TABx, temporal artery biopsy; HSV, herpes simplex virus; HZ, herpes zoster. Age ranges represent ages of subjects by 31 January 2019. * the gender difference was significant at *p* < 0.00001.

## Data Availability

Restrictions apply to the availability of these data. Data was obtained from BC DataScout and are available at https://www.popdata.bc.ca/ with the permission of Population Data BC.
